# Lake Level Fluctuations Boost Toxic Cyanobacterial “Oligotrophic Blooms”

**DOI:** 10.1371/journal.pone.0109526

**Published:** 2014-10-08

**Authors:** Cristiana Callieri, Roberto Bertoni, Mario Contesini, Filippo Bertoni

**Affiliations:** 1 Institute of Ecosystem Study – CNR, Verbania, Italy; 2 Centre for Social Science and Global Health – University of Amsterdam, Amsterdam, The Netherlands; University of Connecticut, United States of America

## Abstract

Global warming has been shown to strongly influence inland water systems, producing noticeable increases in water temperatures. Rising temperatures, especially when combined with widespread nutrient pollution, directly favour the growth of toxic cyanobacteria. Climate changes have also altered natural water level fluctuations increasing the probability of extreme events as dry periods followed by heavy rains. The massive appearance of *Dolichospermum lemmermannii* ( = planktonic *Anabaena*), a toxic species absent from the pelagic zone of the subalpine oligotrophic Lake Maggiore before 2005, could be a consequence of the unusual fluctuations of lake level in recent years. We hypothesized that these fluctuations may favour the cyanobacterium as result of nutrient pulses from the biofilms formed in the littoral zone when the lake level is high. To help verify this, we exposed artificial substrates in the lake, and evaluated their nutrient enrichment and release after desiccation, together with measurements of fluctuations in lake level, precipitation and *D.lemmermannii* population. The highest percentage of P release and the lowest C∶P molar ratio of released nutrients coincided with the summer appearance of the *D.lemmermannii* bloom. The P pulse indicates that fluctuations in level counteract nutrient limitation in this lake and it is suggested that this may apply more widely to other oligotrophic lakes. In view of the predicted increase in water level fluctuations due to climate change, it is important to try to minimize such fluctuations in order to mitigate the occurrence of cyanobacterial blooms.

## Introduction

Cyanobacteria are a phylum of photosynthetic bacteria with a long evolutionary history, dating back to at least ∼3500 million years ago, which allowed them to develop strategic adaptations to conditions of environmental stress [1], [2]. The ability to synthesize UV-absorbing pigments, combined with efficient DNA repair mechanisms and the development of resting structures called akinetes (for most toxic species), protect cyanobacteria from external stressors [2], [3]. The early development of oxygenic photosynthesis – crucial in the atmospheric shift from an anoxic to an oxic Earth – together with phototactic motility and buoyancy control ability give them strong competitive advantages [4]. These adaptations favour their ecological success during periods of environmental change and disturbance, as suggested by their abundance in the geological record in correspondence with mass extinction events [1].

Inland water systems are under increasing pressure from growing human impact and the effects of global warming [5], [6]. In this context, cyanobacterial harmful algal blooms (CHABs) emerged both as indicators of environmental distress and as agents causing positive feedbacks and furthering ecosystemic shifts [7], [8]. The present warming of surface waters and the ensuing thermal stratification positively influence cyanobacteria and have been shown to be, together with nutrient pollution, crucial catalysts for the extension and intensification of CHABs [9], [10], [11], [12]. When a bloom occurs, it increases water turbidity, shading other deeper dwelling populations of producers, thus inhibiting the growth of phytoplankton and macrophytes [9], [13]. These changes in production and habitat cascade to affect consumers in the trophic chain, upsetting the entire system while offering cyanobacteria a positive feedback [10], [14]. These aspects add to the ability of many cyanobacteria to produce toxins to make CHABs a growing threat to freshwater ecosystem services and human health [8]. Hence, the increase in bloom frequency and the expansion of their geographical distribution present an important challenge to water management.

*Dolichospermum lemmermannii* (previously planktonic *Anabaena*, [15]) is one of the species of N-fixing cyanobacteria of the order Nostocales which is experiencing such an expansion. This species also produces hepatotoxic microcystins and neurotoxic anatoxins [16]. The monocyclic heptapeptides microcystins affect animals and also plants, regulating growth and photosynthetic capacity in water plants [17] and inhibiting highly conserved protein phosphatases [18]. In particular *D. lemmermannii* produces the anatoxin-a(*s*) [19] that acts as an acetylcholinesterase inhibitor, exhibiting the lethal power of an insecticide [17], [19]. Though *D. lemmermannii* is characterized by high variability to temperature adaptation, being typical also of cold environments, temperatures between 19 and 26°C have been found to be optimal for most strains [20]. Temperature plays an important role also in the onset of Nostocales akinetes germination in spring and in their growth in summer when they form huge blooms during stratified conditions and calm weather [9], [11]. The expansion in the geographical range of these cyanobacteria follows the predicted connection with freshwater warming, which provides them with a favourable environment [11]. Notwithstanding the extensive evidence for a direct correlation between warming and cyanobacterial blooms (e.g. [1]), though, there is no consensus on any single cause for the blooms. Rather, many factors contribute to the increase in CHABs: among these, temperature and nutrient loading [21] have a crucial influence on the blooms, both directly and indirectly. Nevertheless, considering the difficulties of tackling global warming, most measures to control cyanobacterial blooms focused on nutrient control (e.g. [21]).

This focus on nutrient limitation makes the appearance of *D. lemmermannii* in Lake Maggiore all the more interesting. The lake is part of a larger region of deep subalpine lakes in Northern Italy (which includes lakes Garda, Como, Iseo and Maggiore) progressively colonized by *D. lemmermannii* since the 90s. The altitudinal gradient *D. lemmermannii* followed in colonizing the lakes of the region seems to confirm the connection with the warming of their waters [20]. But, while lakes Como and Iseo are eutrophic lakes characterized by high nutrients concentrations and Lake Garda has been increasingly turning towards a mesotrophic condition, Lake Maggiore is oligotrophic, presenting low nutrient concentration [22], [23]. Cyanobacterial blooms in oligotrophic ecosystems (“oligotrophic blooms”) [24] are not unknown, but to our knowledge were not reported earlier at such scales and have been rather episodic events confined to a restricted zone of the lake. Instead, in Lake Maggiore, *D. lemmermannii* blooms reached the peak of 2×10^6^ cell ml^−1^, covered extended zones on the whole lake surface (Fig. S1), and reoccurred every year since 2005, confirming the chronic colonization of this Nostocales. *D. lemmermannii* was never encountered in Lake Maggiore pelagic station before 2005 [25]. Thus, understanding the mechanisms behind these “oligotrophic blooms” can offer precious insight into how to improve efforts to reduce harmful blooms.

To track the factors that could be favouring the blooms, we documented the pattern of lake level fluctuations, precipitation, average epilimnetic temperatures (0–20 m) and summer *D. lemmermannii* blooms from the beginning of the colonization to 2011 in the oligotrophic, deep Lake Maggiore. Considering the recurrent concomitance of blooms in the aftermath of events of level fluctuations that emerged from these data, we hypothesized a connection between the drying and rewetting of the shore with a pulse of nutrients. To verify this hypothesis and show its connection to summer cyanobacterial blooms in oligotrophic freshwaters we planned in-lake experiments. Over two subsequent years we exposed artificial substrates in Lake Maggiore, quantified the nutrient enrichment (C: carbon, N: nitrogen, P: phosphorus) in the biofilm, simulated the drought and rewetting of the littoral shoreline and measured nutrient release. Here we offer the experimental evidence we gathered and discuss its connection to “oligotrophic blooms”.

## Materials and Methods

### Ethics statement

No specific permission for field activity is required in the location of this research. The sampling activities were not performed in a protected area and they did not involve invertebrates, plant species, corals or fish of any protected species.

### Lake Maggiore and subalpine lake district

Lake Maggiore is among the largest subalpine lakes (212 km^2^, Z_max_ 372 m) in a densely populated area of Northern Italy. Together with lakes Garda, Como, and Iseo it forms one of the largest freshwater resources in Europe, used for agriculture, fisheries, drinking and tourism [26]. It is classified as holo-oligomictic since complete overturn takes place only during periods of strong wind and low air temperatures [27]. The total P concentration decreased from 1977 to 1995 by a factor of 4.6 (Fig. S2) and the lake is now oligotrophic, with TP around 10 *µ*g L^−1^ [22]. The macrophyte vegetation along the 170 km of lake shoreline was sparse when the lake was in mesotrophic conditions [28]. According to a recent survey (CIPAIS 2011, http://www.cipais.org/html/lago-maggiore-pubblicazioni.asp), in 66% of the littoral zone the vegetation is absent or scarce. The synergistic effect of very steep slopes and of largely anthropized coastlines confines the reeds and the macrophytes at the outlet of the main tributaries and at the shallower southern part of the lake.

Since 1980, the lake is included in a monitoring program of biological, chemical and physical parameters, with monthly/fortnightly samplings, along the whole water column (International Commission for Protection of Italian Swiss Waters, CIPAIS) and is included in the Southern Alpine Lakes (LTER) site.

### *D. lemmermannii* bloom, water levels and precipitation

The appearance and development of *D. lemmermannii* was monitored in the pelagic station (www.cipais.org/html/lago-maggiore-pubblicazioni.asp). The bloom appearance and duration along the shoreline was visually inspected, and samples for cyanobacteria enumeration were processed by the Regional Agency for Environmental Protection (ARPA). Lake levels and precipitation are continuously measured at the meteorological station of CNR ISE, operating since 1952.

### Experimental procedure and analyses

**Figure 1:**
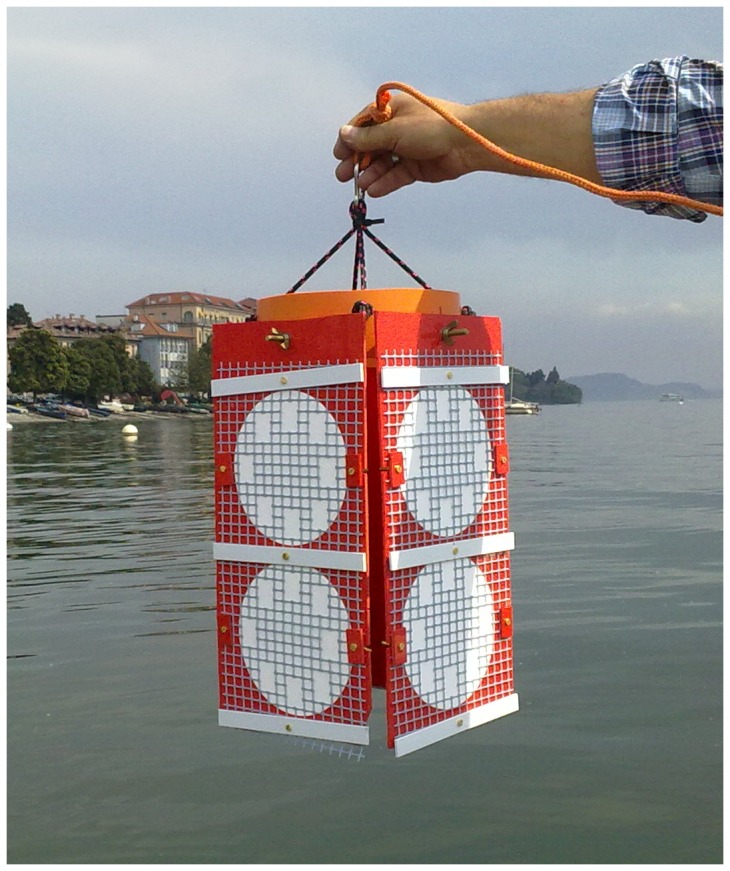
Device used for artificial substrate suspension in Lake Maggiore.

Experiments of littoral substrate simulation were performed in lake Maggiore side arm (Borromeo Basin 45°55′25″N, 8°32′46″E), at a buoy moored 200 m away from the coast (at 50 m bottom depth). During a period of two years (2010–2011) beginning from April up to September/October, thus covering all the productive period, a total of 10 subsequent expositions of artificial substrates were carried out, keeping them submerged at 1.5 m depth for ∼30 days. The substrates were eight glass fiber filters (150 mm diameter Whatman GF/C), previously precombusted at 500°C and placed on the four vertical sides of a standing cage and kept in position by a net (Fig. 1). The vertical position of the filters avoided the collection of material in sedimentation. Upon recovery, two filters were used for the analysis of Particulate Organic Carbon (POC), Particulate Organic Nitrogen (PON) and Particulate Phosphorus (PP), and the other six were used for drying, rewetting and release measurements. From two of the 150 mm glass fiber filters, subsamples were cut with a punch. For POC and PON analyses, ten replicates were run each using a subsample of 191 mm^2^. For PP analyses, five replicates were run each using a subsample of 636 mm^2^.

The remaining six filters were kept in aerated dry boxes at room temperature for the simulation of the drying period, when the lake level lowered and the shore was covered by the dried up deposited material. The desiccation phase lasted 30 days. After desiccation, each filter was placed in glass, nutrient decontaminated, containers (washed with acid and MQ water) and covered by 200 ml of MQ water. This operation simulates the increase of lake level that submerges the shore after heavy rain. After 3 days, the water covering the filters in the containers was analysed (three replicates) to measure the release of total organic carbon (TOC) and phosphorus (TP). The maximum release was always obtained after 3 days, as verified by measuring the release 30 days after the rewetting and compared with the 3-days release (data not shown). The POC and PON on the filters were determined by high temperature oxidation and thermal conductivity detector using Elemental Analyser FlashEATM 1112 (CHN) [29]. PP and TP were analysed with the ammonium molybdate/potassium persulphate classical method following A.P.H.A [30]. TOC was measured with a Shimadzu analyser (5000A).

### Calculations and statistics

All the expositions were standardized to a period of 30 days both for accumulated and released nutrients and data reported as per unit area (m^2^), per month. The amount of nutrients accumulated/released along the entire shoreline (170 km) was computed assuming a lake level variation of one meter, a shore slope of 45°, and a rugosity index of the shore similar to that of granite as the bedrock of the lake is mainly granitic.

To calculate the monthly accumulation/release (in kg) we used the following formula:
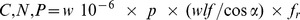
where:

*w* is the weight of C, N, P from the artificial substrates per unit area (mg m^2^)

*p* is the lake perimeter (in our case 170 10^3^ m)

*wlf* is the water level fluctuation (we assumed 1 m)

*α* is the shoreline steepness (we assumed 45°)

*f_r_* is the rugosity index of shoreline, which increases the geometric surface depending on the surface roughness; (here we used 2.99, the *f_r_* of granite [31]).

We decided to keep the substrate protected from the atmospheric events to standardize the different exposition periods, being aware that, in this case, we could not test any effects due to UV or high PAR exposition of the substrates.

Trends in the long-term data series were verified using Mann-Kendall trend test. P values of the statistical tests were related to a significance value of α = 0.05. The null hypothesis states that there is no trend in the data series, whereas the alternative hypothesis states that there is a trend. In cases with P values lower than α = 0.05, the null hypothesis was rejected. All analyses were carried out with the Microsoft EXCEL add-in programs XLSTAT 2012-TIME. Contour map of temperature was performed by SURFER 10 (Golden software) using a kriging algorithm.

## Results

### Temperature, lake level, precipitation and cyanobacteria bloom

**Figure 2:**
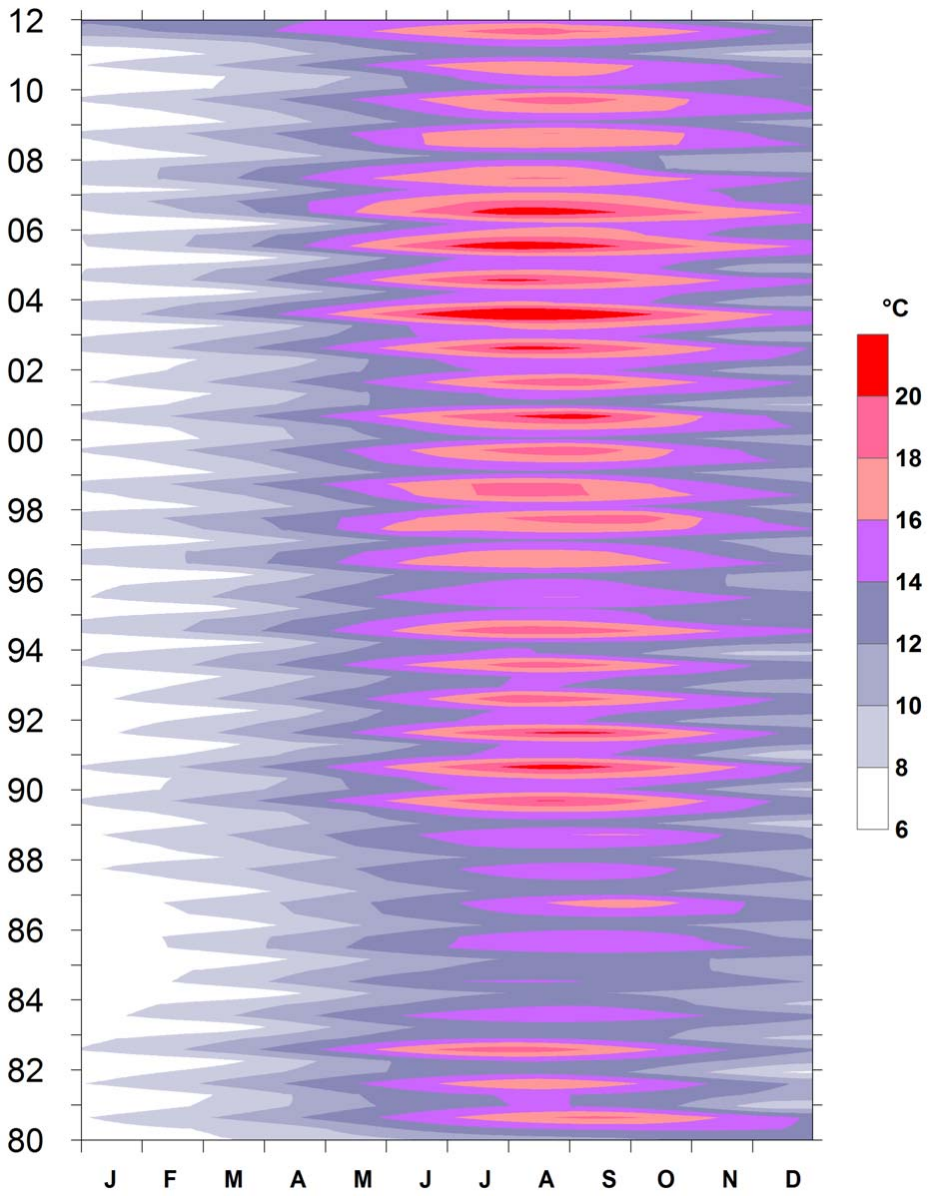
Epilimnetic temperature contour map (0–20 m, °C, 346 data) of Lake Maggiore, 1980–2011.

The 30-year data set of continuous epilimnetic temperature records in Lake Maggiore shows a significant trend (Mann-Kendall/Two tailed trend test, n: 346, p<0.01) toward warming (Fig. 2). Besides the increase of temperature, there was an extension of the higher temperature along the season. The number of days of mean epilimnetic temperature above 16°C increased significantly from 1980 to 2011 (Mann-Kendall/Two tailed trend test, n: 32, p<0.0001).

**Figure 3:**
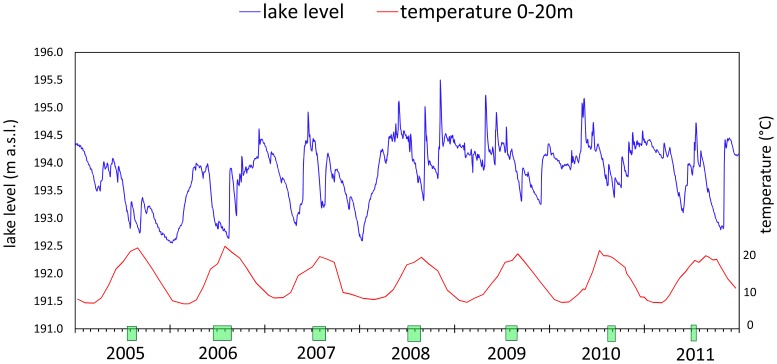
Daily average lake levels (blue line) and monthly average epilimnetic temperature (red line) of Lake Maggiore (2005–2011). The green bars on the X-axis indicate *D. lemmermannii* period of occurrence.

**Figure 4:**
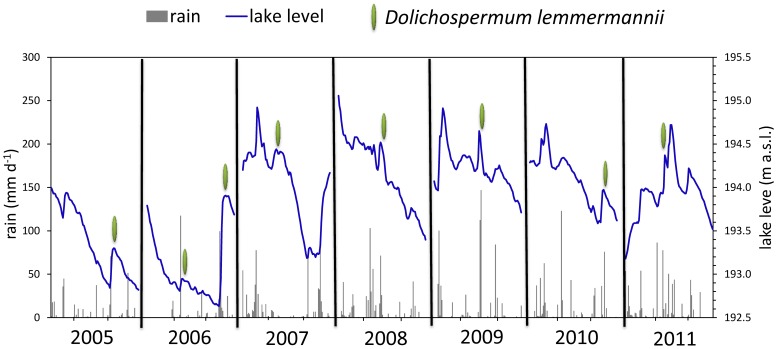
Lake level, precipitation and *D. lemmermannii* bloom detailed for June, July and August in Lake Maggiore (2005–2011). Green symbol = *D. lemmermannii* bloom peaks; blue line = lake level variations; gray histograms = precipitation.

Plotting the data of the daily average lake levels and monthly temperatures from 2005, year of the *D.lemmermannii* appearance, to 2011 we marked on the X-axis the *D.lemmermannii* period of occurrence (Fig. 3). Firstly, we noted that the bloom always occurred in summer time, as expected, and at an epilimnetic temperature between 15 and 20°C. Thus, we focused on analysing lake levels, precipitation and blooms in the three summer months during which *D.lemmermannii* appeared regularly in Lake Maggiore (Fig. 4). We noticed that the peak of the bloom (marked with a symbol in Fig. 4) appeared after a sudden increase of lake level following a precipitation event. In 2005, *D.lemmermannii* reached the peak of 2×10^6^ cell ml^−1^ and covered extensive zones over the whole lake surface (Fig. S1) [32]. Its presence was not related to the absolute value of the lake level, rather the peaks of the bloom coincided with water level increases mainly after a strong precipitation. The bloom density and extension was different in the seven years and declined from 2005 to 2009 to increase again in 2010 and 2011 (Fig. S1). It should be noted that in the two years of maximum abundance of *D.lemmermannii* (2005–2006) there had been a very dry summer with low lake level. In the first year of appearance (2005), *D.lemmermannii* bloomed in August, after a lake level increase of 40 cm in 7 days due to 70 mm d^−1^ rain. In the summer of the following year *D.lemmermannii* bloomed twice: in July, after a heavy rain (117 mm d^−1^), and in August (99 mm d^−1^). (Lake level increase in Lake Maggiore is not related linearly to precipitation intensity as the level is regulated at the outlet to match downstream water requirements and flood control).

### Artificial substrates experiments

The nutrients on the artificial substrates showed pronounced seasonal changes with the highest values reached in spring for C and N and in autumn for P. The nutrients measured monthly ranged from 618 to 3066 mg C m^−2^, 136–511 mg N m^−2^, and 3.78–13.4 mg P m^−2^ (Table 1). On average 1782 mg C m^−2^, 295 mg N m^−2^ and 9.2 mg P m^−2^ were present in the biofilm developed on substrates, in one month. Similarly, in terms of kilos produced in one month on 1 meter of shoreline (170 km), the highest amount of C and N was in spring (2206 kg and 368 kg) and that of P in late summer (9.66 kg). The quality of the material deposited changed along the season as demonstrated by the C∶P and C∶N molar ratio (Table 1).

**Table 1:** Average and standard deviation (10 replicates) of the estimated organic carbon (C), nitrogen (N) and phosphorus (P) per square meter per month present on the biofilm formed on the artificial substrates (first three columns).

		mgC m^−2^ (av±sd)		mgN m^−2^ (av±sd)		mgP m^−2^ (av±sd)		kg C (av±sd)		kg N (av±sd)		kg P (av±sd)		C∶P	C∶N
**2010**	26Apr–21Jun	2348	421	286	65.7	3.78	0.41	1688	303	206	47.2	2.72	0.29	621	8.20
	24Jun–22Jul	3066	489	511	96.2	11.1	2.55	2206	352	368	69.2	7.99	1.83	276	6.00
	**27Jul–25Aug**	**2221**	**358**	**297**	**40.7**	**11.2**	**0.74**	**1596**	**257**	**214**	**29.3**	**8.07**	**0.53**	**198**	**7.47**
	25Aug–22Sep	2008	184	261	25.1	13.4	1.23	1443	132	187	18.0	9.66	0.88	149	7.70
**2011**	14Apr–11May	618	70.8	136	73.9	6.60	1.41	444	50.9	97.8	53.1	4.75	1.01	93.6	4.54
	12May–8Jun	2867	246	455	25.4	8.04	1.41	2060	177	327	18.3	5.78	1.01	356	6.31
	**9Jun–11Jul**	**772**	**143**	**190**	**31.6**	**8.82**	**1.99**	**555**	**103**	**137**	**22.7**	**6.34**	**1.43**	**87.6**	**4.06**
	13Jul–9Aug	1028	208	242	34.5	8.24	1.50	739	150	174	24.8	5.93	1.08	125	4.24
	9Aug–5Sep	938	203	265	65.0	12.9	2.97	674	146	191	46.7	9.25	2.14	72.8	3.53
	5Sep–11Oct	1957	226	305	31.8	8.17	1.24	1407	162	219	22.9	5.87	0.89	239	6.42

Kilos of C, N, P estimated to be on Lake Maggiore shoreline (170 km) after a level variation of 1 m, in one month, assuming a 45° shore slope and rugosity index of granite. C∶P and C∶N molar ratios are reported. The periods of the appearance of the *D. lemmermannii* bloom are in bold.

The average estimated quantity of C and P released from the substrates after desiccation and rewetting followed patterns similar to those of the material on the substrates in spring, but was different in late summer both for C and for P (Table 2). The nutrient release after rewetting ranged 22–202 mg C m^−2^ and 1.5–25.2 mg P m^−2^ (Table 2). In terms of kilos released in one month from 1 meter of shoreline (170 km), the highest C release (145 kg) occurred in June/July 2010, while the highest P release (18 kg) took place in July/August 2010, in correspondence with the period of cyanobacteria bloom.

**Table 2:** Average and standard deviation (3 replicates) of the estimated organic carbon (C), and phosphorus (P) released after desiccation and rewetting, per surface filter per day (first two columns).

		mgC m^−2^ (av±sd)		mgP m^−2^ (av±sd)		kg C (av±sd)		kg P (av±sd)	
**2010**	26Apr–21Jun	156	24.1	4.92	0.86	112	17.3	3.54	0.62
	24Jun–22Jul	202	33.7	15.6	0.82	145	24.2	11.2	0.59
	**27Jul–25Aug**	**97.6**	**3.25**	**25.2**	**8.60**	**70.1**	**2.33**	**18.1**	**6.18**
	25Aug–22Sep	105	8.19	5.04	1.16	75.6	5.89	3.62	0.83
**2011**	14Apr–11May	48.9	7.09	1.51	0.23	35.1	5.09	1.09	0.16
	12May–8Jun	149	18.1	3.69	1.16	107	13.0	2.66	0.83
	**9Jun–11Jul**	**51.7**	**9.58**	**6.02**	**1.61**	**37.2**	**6.88**	**4.33**	**1.16**
	13Jul–9Aug	22.4	3.67	3.83	1.43	16.1	2.64	2.75	1.03
	9Aug–5Sep	38.1	5.16	2.52	0.23	27.4	3.71	1.81	0.17
	5Sep–11Oct	119	13.9	3.15	0.51	85.7	10.0	2.27	0.36

Kilos of C and P released from Lake Maggiore shoreline (170 km) after a level variation of 1 m, in one month, assuming a 45° shore slope and rugosity index of granite. The periods of the appearance of the *D. lemmermannii* bloom are in bold.

**Figure 5:**
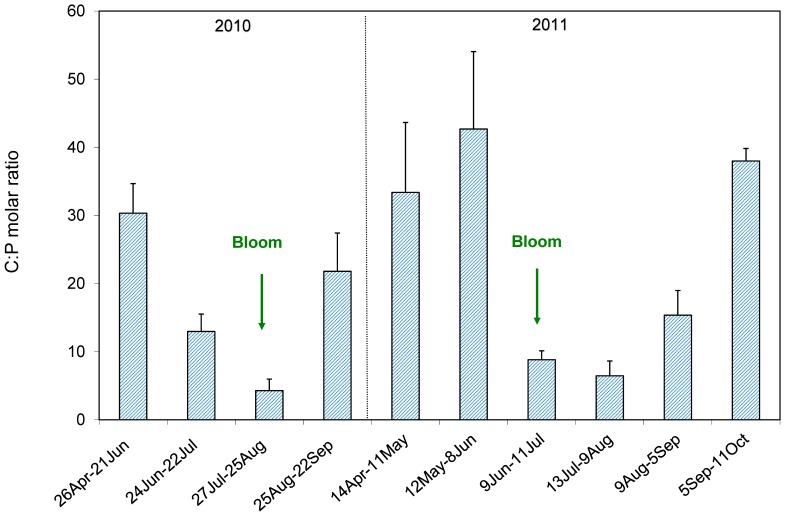
C∶P (carbon: phosphorus) molar ratio of the released material from the artificial substrates. The histograms represent the mean ratios (±standard deviation) obtained in the experiments done in 2010 and 2011. The green arrows indicate the periods in which the bloom occurred.

The C∶P molar ratio variations estimated with the artificial substrates experimentation show the lower values in correspondence of the bloom in the lake (Fig. 5). Therefore, the highest release of P was in coincidence with the cyanobacteria bloom in the exposition periods 27 July–25 August 2010 and 9 June–11 July 2011. Similarly, the percentage of nutrient release reached their maximum at the bloom and on average was 5% for C and 77% for P.

### Discussion

Bloom drivers, a recent study showed [33], depend on the cyanobacterial taxon and on the lake trophic state; in mesotrophic lakes, temperature is a better predictor of Nostocales blooms. In Lake Garda, where the trophic status has increased to mesotrophic conditions, with P concentrations almost doubled in 40 years [26], warming temperatures and an exogenous introduction of cyanobacteria are likely at the origin of *D. lemmermannii* appearance. Similarly, climate change induced warming could explain the colonization of the eutrophic lakes Como and Iseo. Conversely, in low nutrient conditions exogenous nutrient loading seems to be the most effective driver of the intensification of high biomass cyanobacterial bloom [33]. Therefore, even if the 32-year data set of continuous epilimnetic temperature records in Lake Maggiore confirms the significant trend toward warming, the P loading source remained an open question. In fact, in Lake Maggiore no obvious nonpoint source nutrient pollution can be found. Furthermore, the lake is deep and, in the last thirty years, the mixing depth never exceeded 200 m [34], therefore a P recirculation from the sediment as a cause of the cyanobacteria bloom is unlikely.

Having ruled out P resuspension from the deep sediments, we tested the hypothesis of a release from the littoral zone. Our data set of daily average lake levels and monthly average temperature from 2005 to 2011 showed that the blooms occurred at summer temperatures and in particular, focusing on the summer months, a synchrony between the blooms and episodes of water level fluctuations emerged. A connection between water level fluctuations and the release of nutrients from the littoral zone had already been hypothesized [9], [21], [35], but proved hard to quantify and understand.

Through our experiments with artificial substrates, we demonstrate a variation of the C∶P molar ratio during the two years of study. In both years, the peak of *D. lemmermannii* (23 August 2010 and 30 June 2011) occurred after a rapid increase of lake level following a period of drought, as was observed since 2005. The data from the artificial substrates indicate that bloom formation corresponded to the release of material with a low C∶P molar ratio and a high percentage of phosphorus released from the shore. These conditions are explained by the release of previously accumulated phosphate and the higher mineralization of organic phosphorus by activation of phosphatase [36] that follows drought and rewetting events. Thus, desiccation and rewetting lead to increased availability of P in the littoral zone, potentially triggering cyanobacteria growth also in oligotrophic systems. Even though other abiotic factors like high temperature, CO_2_ and underwater light conditions influence cyanobacterial blooms, here we show that lake level fluctuations have a crucial role in *D. lemmermannii* colonization of oligotrophic deep lakes, like Lake Maggiore. We are aware that also biotic interactions can have a role in cyanobacterial bloom. In particular, the submerged macrophytes can be light-limited by the increased water level and consistently the cyanobacterial bloom favoured. Likely, this interaction can only be of local importance in this deep lake, whose steep littoral slope supports a low submerged macrophyte biomass.

The amount of nutrients present on the biofilm formed on the artificial substrates fluctuated along the year in accordance with the seasonal pattern of autochthonous particulate organic matter production, reflecting the population successions occurring in the lake. We concomitantly observed variations of the C∶P and C∶N molar ratio, which indicate a change in the quality of the biofilm on the substrates. The maximum amount of carbon (2206 kg) occurs in spring and that of phosphorus (9.7 kg) in late summer. Considering that 10 kg of elemental phosphorus correspond approximately to 80 kg of monocalcium phosphate, a common fertilizer, this quantity of P amounts to a noteworthy nonpoint nutrient source from the shore, particularly in an oligotrophic lake. Moreover, our estimate of P on the substrates does not include the organic phosphorus present in the microorganisms that could be, successively, either directly used by cyanobacteria through phosphatase activity, or mineralized by microorganisms [36].

The maximum release of nutrients was 145 kg of C and 18.1 kg of P, in 1 month, on lake perimeter, with 1 m lake level fluctuation. Even if the absolute amount released was higher for C than for P, the relative release was 5% for C and 77% for P (two years average). The percentage of P released fluctuated more than C in the two years, and reached the maximum in coincidence with the cyanobacteria bloom. The contribution of the P released from the shore was 53 mg P m^−2^ in 2010 and 20 mg P m^−2^ in 2011. These estimates are not far from the allochthonous P input as areal contribution from Lake Maggiore rivers which are 22 and 26 mg P m^−2^ y^−1^ in 2010 and 2011, respectively [22]. Nevertheless, it must be emphasized that river input in the lake is a point-source and the nutrient load quickly sinks in the water column due to temperature differential between lake and colder river waters. On the other hand, the contribution from the shore is a non-point-source, which remains near the surface layers.

Over the last decade, we assisted to drought-induced decreases in lake-level, frequently followed by heavy precipitation due to extreme meteorological events. In light of this, the pulse of P from the shore drying and rewetting cannot be ignored, particularly in an oligotrophic lake.

In conclusion, our experiments show the important role of nutrient release from the drying and rewetting of lake shores in the expansion of cyanobacteria in oligotrophic lakes. Fluctuations in water levels seem to be increasing as an adverse effect of climate change, suggesting that nutrient release from the littoral zone will gain a growing impact on freshwater ecosystems [35]. Nevertheless, changes in water levels also offer a different ground for water management interventions. In fact, most central European lakes have been largely regulated since long time and water levels are thus often amenable to external control [35]. For this reason, highlighting the mechanisms that relate water level fluctuations, nutrient pulses and cyanobacterial blooms promises a better handle on possible measures to employ in limiting the spread of CHABs and thus preserve fundamental freshwater ecosystems and resources.

## Supporting Information

Figure S1 ***D. lemmermannii*** **bloom quantification and extension.** (a) Maxima of *D. lemmermannii* during bloom (2005–2011) redrawn from the report by the Regional Agency for Environmental Protection. The counting has been done at different littoral stations of Lake Maggiore. The bloom appeared regularly every summer since 2005 but its density decreased in the following years except in 2010 when the cyanobacterial number reached again the level of moderate probability of adverse health effects (10^5^ cell ml^−1^, according to the WHO Guideline 2003). (b) Satellite pictures of surface algal bloom onset, from July 20 to July28, 2005. Higher IR/R ratio corresponds to surface bloom areas. Data from the MERIS Full Resolution images available from non-cloudy days, kindly provided by Giardino C., CNR IREA.
(DOCX)

Figure S2 **Long-term trend of the main nutrients in Lago Maggiore from 1956 to 2011.** (a) Total phosphorus (TP) and reactive phosphorus (RP), (b) nitrate-N (N-NO^3^) and total nitrogen (TN). The period of eutrophication from 1970 to 1977 and of re-oligotrophication from 1977 to 1995 are apparent. After 1995 a period of P stabilization followed. Nitrate-N shows an increasing trend particularly from 1955 to 1975. *D. lemmermannii* appeared in years of well-defined oligotrophic conditions. Data kindly provided by Mosello R. and Rogora M. published online in the CIPAIS reports and updated to 2011.
(DOCX)

Dataset S1 **Experimental data summary.**
(XLS)
