# Supplementary material for: Lake Level Fluctuations Boost Toxic Cyanobacterial “Oligotrophic Blooms”
Source: PLoS One. 2014 Oct 8;9(10):e109526. doi: 10.1371/journal.pone.0109526 (PMC4190310; doi:10.1371/journal.pone.0109526)
Supplement: Figure S1 — D. lemmermannii bloom quantification and extension. (a) Maxima of D. lemmermannii during bloom (2005–2011) redrawn from the report by the Regional Agency for Environmental Protection. The counting has been done at different littoral stations of Lake Maggiore. The bloom appeared regularly every summer since 2005 but its density decreased in the following years except in 2010 when the cyanobacterial number reached again the level of moderate probability of adverse health effects (105 cell ml−1, according to the WHO Guideline 2003). (b) Satellite pictures of surface algal bloom onset, from July 20 to July28, 2005. Higher IR/R ratio corresponds to surface bloom areas. Data from the MERIS Full Resolution images available from non-cloudy days, kindly provided by Giardino C., CNR IREA. (DOCX) [file pone.0109526.s001.docx]

**(a)**


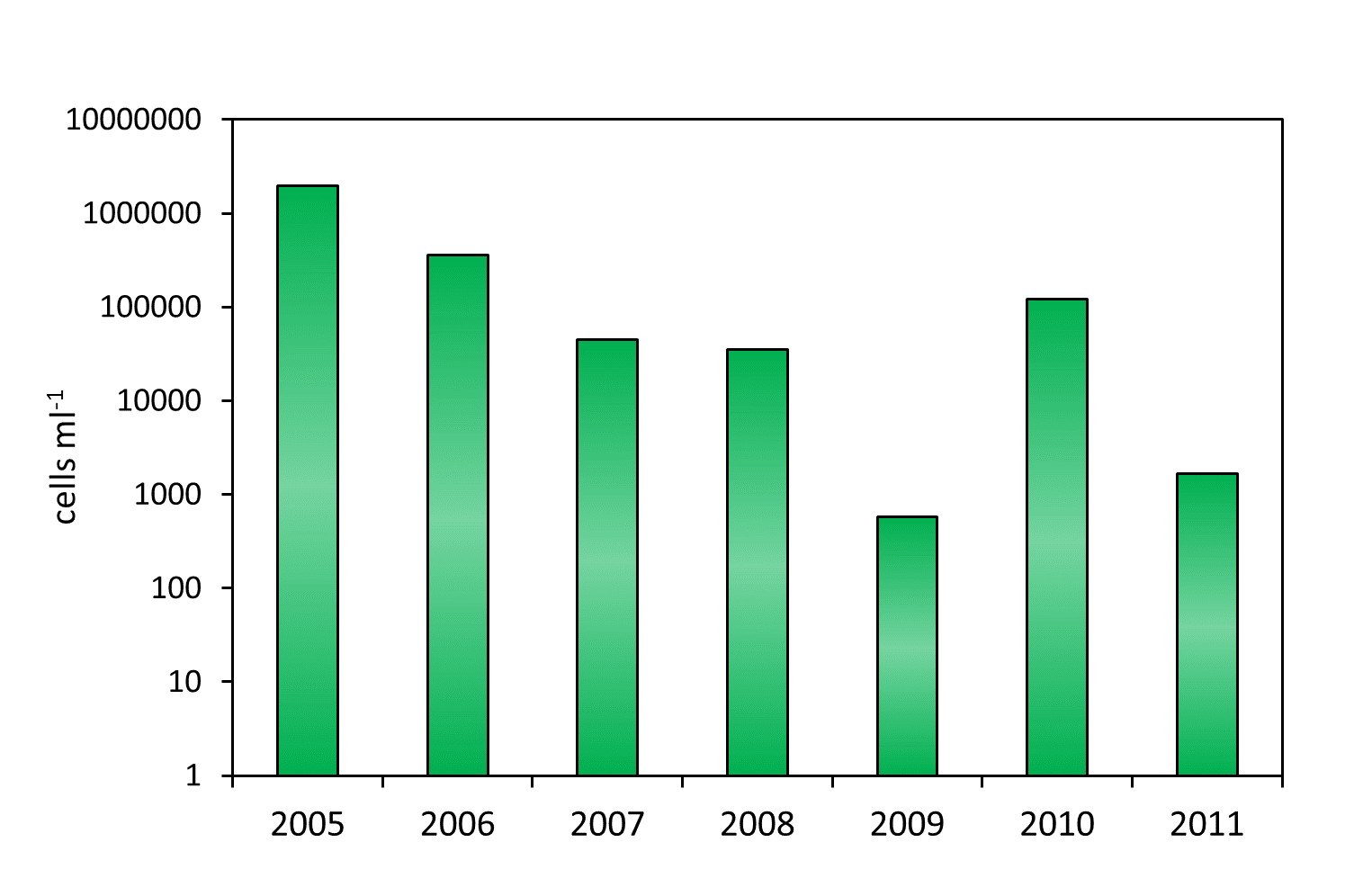


**(b)**

**Figure S1.** ***D. lemmermannii* bloom quantification and extension.** (a) Maxima of *D. lemmermannii* during bloom (2005-2011) redrawn from the report by the Regional Agency for Environmental Protection. The counting has been done at different littoral stations of Lake Maggiore. The bloom appeared regularly every summer since 2005 but its density decreased in the following years except in 2010 when the cyanobacterial number reached again the level of moderate probability of adverse health effects (10^5^ cell ml^-1^, according to the WHO Guideline 2003). (b) Satellite pictures of surface algal bloom onset, from July 20 to July28, 2005. Higher IR/R ratio corresponds to surface bloom areas. Data from the MERIS Full Resolution images available from non-cloudy days, kindly provided by Giardino C., CNR IREA.
