# Supplementary material for: Lake Level Fluctuations Boost Toxic Cyanobacterial “Oligotrophic Blooms”
Source: PLoS One. 2014 Oct 8;9(10):e109526. doi: 10.1371/journal.pone.0109526 (PMC4190310; doi:10.1371/journal.pone.0109526)
Supplement: Figure S2 — Long-term trend of the main nutrients in Lago Maggiore from 1956 to 2011. (a) Total phosphorus (TP) and reactive phosphorus (RP), (b) nitrate-N (N-NO3) and total nitrogen (TN). The period of eutrophication from 1970 to 1977 and of re-oligotrophication from 1977 to 1995 are apparent. After 1995 a period of P stabilization followed. Nitrate-N shows an increasing trend particularly from 1955 to 1975. D. lemmermannii appeared in years of well-defined oligotrophic conditions. Data kindly provided by Mosello R. and Rogora M. published online in the CIPAIS reports and updated to 2011. (DOCX) [file pone.0109526.s002.docx]

**(a)**


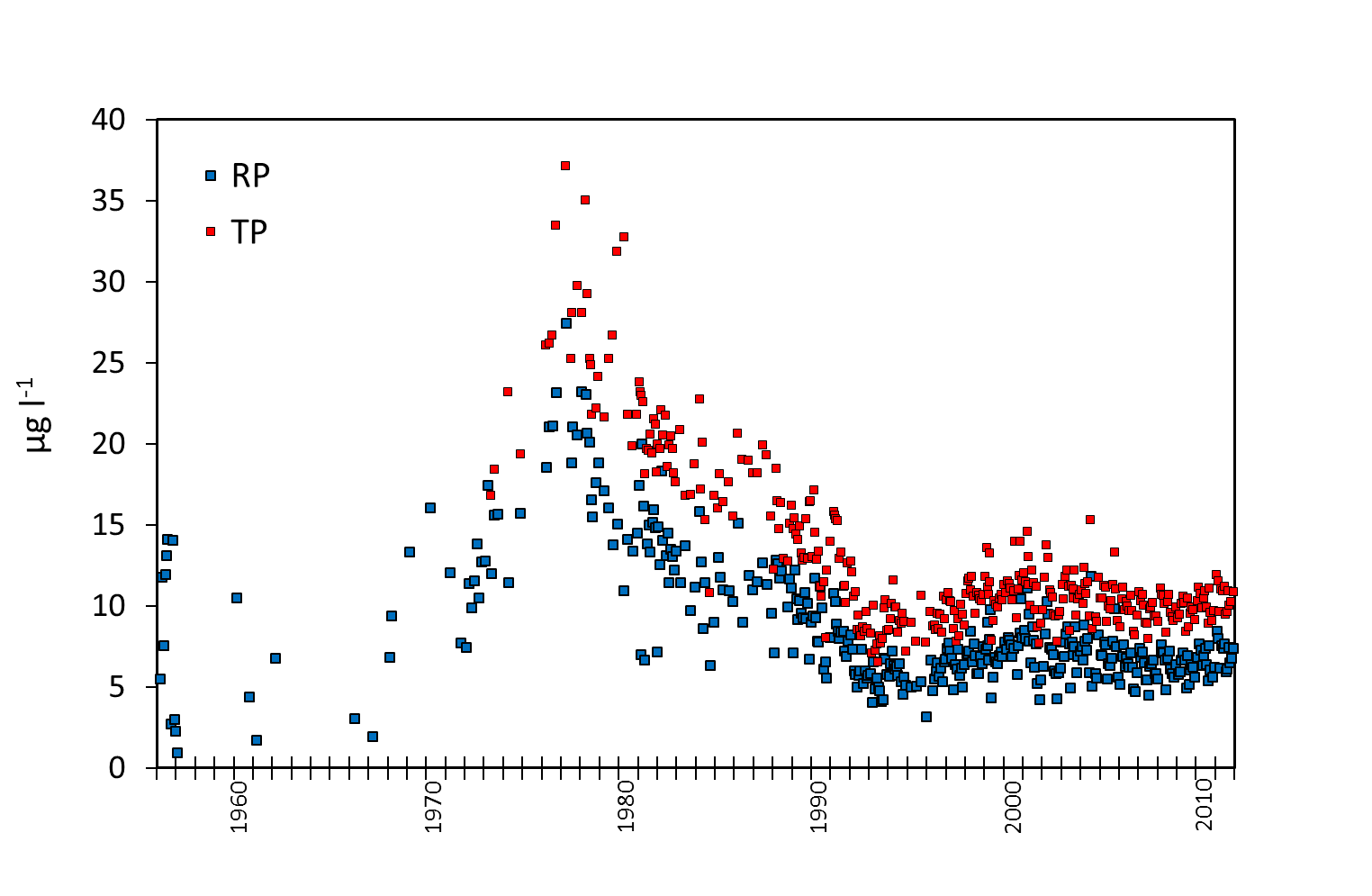


**(b)**


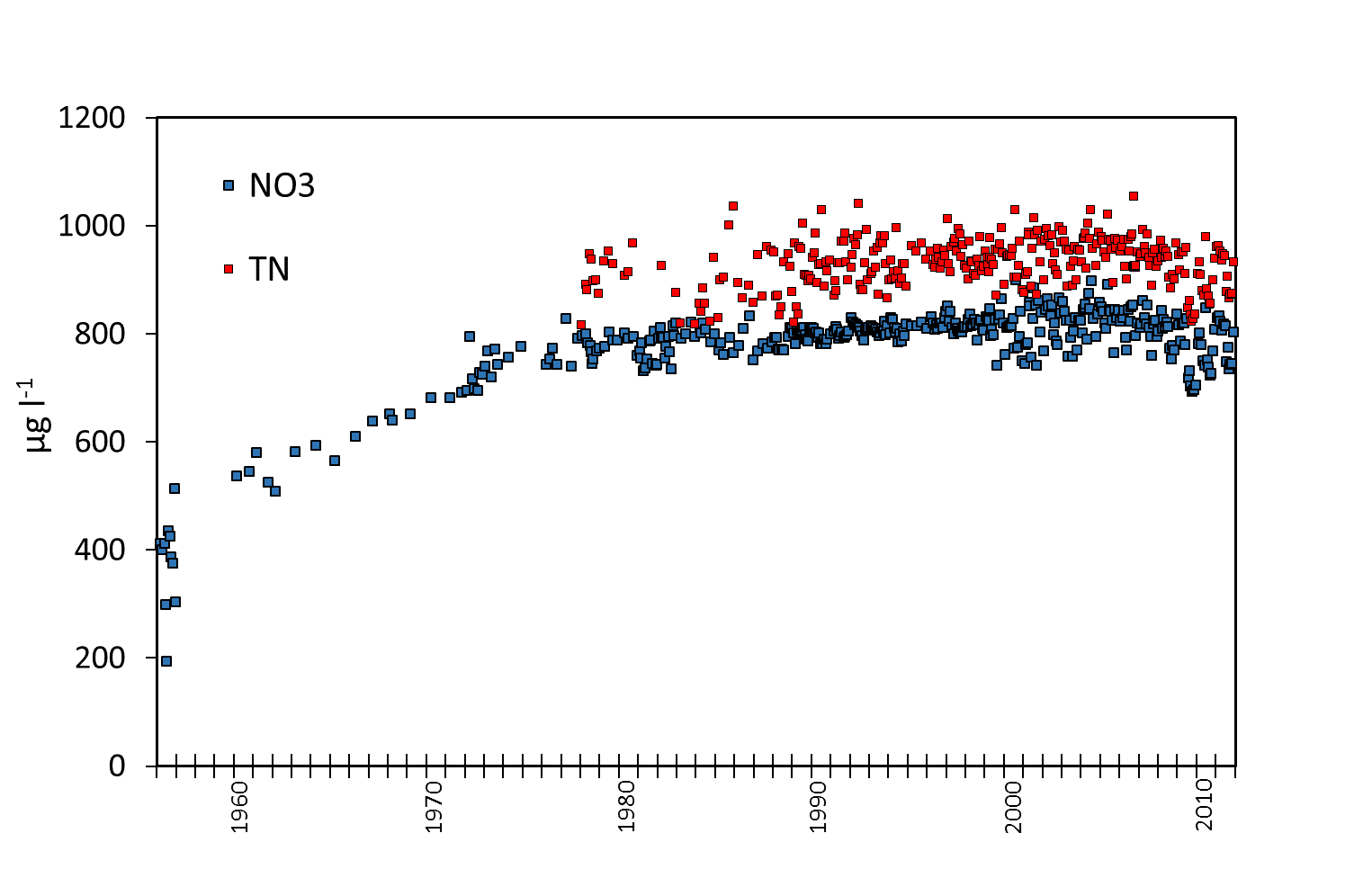


**Figure S2.** **Long-term trend of the main nutrients in Lago Maggiore from 1956 to 2011.** (a) Total phosphorus (TP) and reactive phosphorus (RP), (b) nitrate-N (N-NO^3^) and total nitrogen (TN). The period of eutrophication from 1970 to 1977 and of re-oligotrophication from 1977 to 1995 are apparent. After 1995 a period of P stabilization followed. Nitrate-N shows an increasing trend particularly from 1955 to 1975. *D. lemmermannii* appeared in years of well-defined oligotrophic conditions. Data kindly provided by Mosello R. and Rogora M. published online in the CIPAIS reports and updated to 2011.
